# Utilization of a Smart Sock for the Remote Monitoring of Patients With Peripheral Neuropathy: Cross-sectional Study of a Real-world Registry

**DOI:** 10.2196/32934

**Published:** 2022-03-01

**Authors:** Henk Jan Scholten, Chia-Ding Shih, Ran Ma, Kara Malhotra, Alexander M Reyzelman

**Affiliations:** 1 Siren Care, Inc San Francisco, CA United States; 2 California School of Podiatric Medicine Samuel Merritt University San Francisco, CA United States

**Keywords:** diabetes, diabetic foot ulcer, temperature monitoring, digital health, wearable, neuropathy, remote patient monitoring, ulcer, foot, temperature, monitoring, medical device, utilization, risk, complication, registry

## Abstract

**Background:**

Remote patient monitoring (RPM) devices are increasingly being used in caring for patients to reduce risks of complications. Temperature monitoring specifically has been shown in previous studies to provide a useful signal of inflammation that may help prevent foot ulcers.

**Objective:**

In this cross-sectional study, we evaluated utilization data for patients who were prescribed smart socks as remote temperature monitoring devices.

**Methods:**

This study evaluated data from a patient registry from January to July 2021. The utilization data, which were collected starting from the first full month since patients were prescribed the smart socks, were evaluated along with retention over time, the average time that the socks were worn, and the number of days that the socks were worn per month and per week.

**Results:**

A total of 160 patients wore the smart sock RPM device for 22 to 25 days per month on average. The retention rate was 91.9% (147/160) at the end of the 7-month period; a total of 13 patients were lost to follow-up during this period. The average number of days that the socks were worn per week was 5.8. The percentage of patients with a utilization rate of >15 days ranged from 79.7% (106/133) to 91.9% (125/136) each month.

**Conclusions:**

This study shows a high level of utilization for a smart sock RPM device and a high compliance rate. A future prospective study on the clinical outcomes after the use of the smart socks may further solidify the idea of conducting temperature monitoring for foot ulcer prevention.

## Introduction

Remote patient monitoring (RPM) has emerged as a critical method in disease prevention. A wide range of devices are designed to monitor physiologic indicators of clinical interest for a variety of health conditions, especially diabetes and related complications. Studies have demonstrated the efficacy and promise of RPM in diabetes management. Su et al [1] examined glycemic control for 1354 patients and concluded that patients who underwent more frequent and regular remote monitoring had lower hemoglobin A_1c_ levels than those of patients with lower adherence to the program. Further, the willingness to adopt remote monitoring was evaluated in 1577 patients with diabetes, and perceived intrusiveness was a main factor for whether a patient would adopt monitoring in diabetes management [2].

Diabetic foot ulcers (DFUs) are a highly prevalent complication for people living with diabetes, who have an estimated 25% lifetime risk of developing DFUs [3]. Temperature was first identified as a predictive factor for ulceration by Benbow et al [4]. Researchers further developed temperature monitoring by measuring multiple sites on each foot to assess temperature differentials that may predict the onset of a neuropathic ulceration [5]. “Areas that are likely to ulcerate have been associated with increased local skin temperatures due to inflammation and enzymatic autolysis of tissue” [6]. Identifying areas of injury through inflammation tracking allows patients and their providers to intervene to reduce inflammation before a wound develops [6].

Various technologies have been developed that use temperature differentials to remotely monitor diabetic foot health [7]. One technology is a smart sock that can be worn by patients and has a regular connection to the cloud for the capture and sharing of temperature data with health care professionals. The socks have temperature sensors embedded inside of the fabric, so that it is soft and comfortable for the user. The patient data are monitored for an elevation in temperature that indicates inflammation—an early sign of wound formation. Although the socks are mainly intended for preventative therapy, this product has also been used to track the course of developing ulcers. Additionally, the socks can be used by people who have undergone an amputation; the socks use an ipsilateral temperature algorithm to detect temperature elevations in 1 foot. Reyzelman et al [8] first evaluated the smart socks in a 35-patient study; patients reported that the socks were easy to use and comfortable, ranking them with a median score of 9 and 10 for comfort and ease of use, respectively, on a 10-point scale. These ratings for ease of use and comfort indicate potentially low intrusiveness and a high willingness to use smart socks as an RPM device.

In this cross-sectional study, we reviewed real-world data from patients using a smart sock temperature monitoring device (Siren Socks; Siren Care) to assess compliance and utilization levels. The purpose of this study is to determine how patients adhere to an RPM program for DFU prevention that involves the use of smart socks.

## Methods

### Study Design

Patients in the Siren Care registry who were enrolled throughout July 2021 were retrospectively reviewed. This registry is an institutional review board (IRB)–approved protocol (IRB submission title: *Temperature and Activity Data from the “Siren Socks and Foot Monitoring System” – A Multicenter Post Market Registry Study with Retrospective and Prospective Analysis*; WCG IRB study number: 1284366). The qualification for subscribing to the smart socks was the diagnosis of peripheral neuropathy. The inclusion criteria were patients using the smart sock temperature monitoring device for at least 1 day, those who were at participating sites, and those who consented to the registry, as per the IRB-approved protocol. The first calendar month of utilization was excluded due to the variability in which days of the month patients began using the RPM device. The utilization data were measured as the amount of time that the socks were worn, as measured by the smart sock device, in terms of wear time per day as well as the number of days that the socks were worn per month and per week.

### Description of the Temperature Monitoring Device

**Figure 1:**
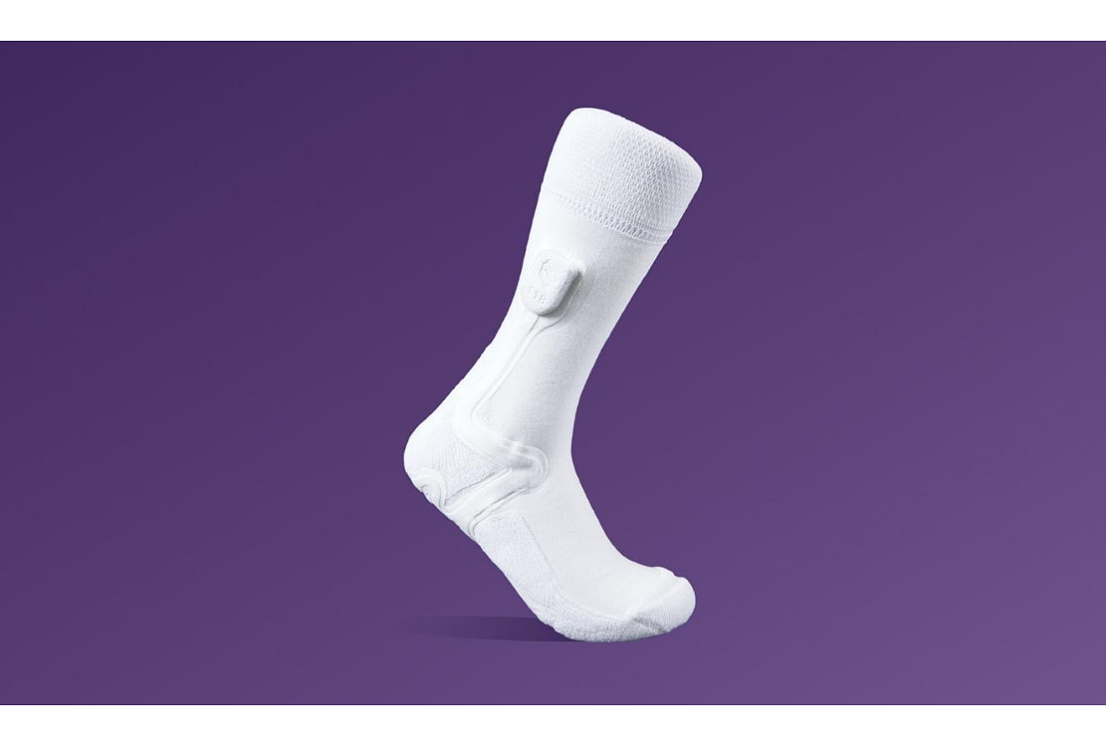
The smart sock remote temperature monitoring device (Siren Socks; Siren Care).

The smart sock device takes continuous measurements of temperature at 6 points on each foot (the hallux, the heel, the arch, metatarsal 1, metatarsal 3, and metatarsal 5). The temperature is measured automatically throughout the day. The socks turn on upon wear and turn off automatically when they are no longer worn. No charging is necessary by the patient. No smartphone is necessary to be used by the patient for data transmission. A hub is plugged into the wall for data transmission, and monitoring data are also stored on the socks to allow for monitoring when patients are away from home. The socks are designed to be machine washable and can be reused for a period of up to 1 year (Figure 1). The socks’ lifetime is around 1 year, but this can be longer depending on usage. Socks are replaced after normal wear and tear. Data were collected and monitored by prescribing physicians and their designated staff. Any temperature differentials greater than 2.2 °C resulted in an alert to a monitoring nurse that required follow-up via a phone call to the patient.

### Description of Outcome Measures

The primary outcome measure was the number of days that the socks were worn per month and per week. The secondary outcome measure was the amount of time that the socks were worn per day.

### Statistical Analysis

Descriptive statistics were performed, and the analysis was performed by using Microsoft Excel (version 16.51; Microsoft Corporation).

### Ethics Approval

This study was approved by the WCG IRB (study number: 12843666). If an individual wished to participate in the study, they were informed about the study objectives, and they could consent through a mobile app or over the phone after having started using the socks.

## Results

A total of 160 patients met the inclusion criteria as of July 2021. Data from previous months of enrollment were included, beginning in January 2021 (Table 1).

**Table 1:** Smart sock utilization (>15 days and >5 days of wear).

Utilization characteristics	January 2021	February 2021	March 2021	April 2021	May 2021	June 2021	July 2021
Active patients, n	10	20	23	62	110	133	136
Patients with >15 days of wear, n (%)	8 (80)	17 (85)	20 (87)	55 (89)	95 (86)	106 (80)	125 (92)
Patients with >5 days of wear, n (%)	10 (100)	20 (100)	22 (96)	60 (97)	108 (98)	125 (94)	132 (97)

Across the entire studied period, patients wore the socks for 5.8 (SD 1.7) days per week on average. The median number of days that the socks were worn was 7 days per week. Further, 93% of the time, socks were worn for at least 3 days per week (Table 2).

**Table 2:** Smart sock utilization (number of days worn per month and week and number of hours worn per day).

Utilization characteristics	January 2021	February 2021	March 2021	April 2021	May 2021	June 2021	July 2021
Number of wear days per month, mean (SD; median)	23.0 (7.8; 26)	23.1 (7.8; 27)	24.8 (8.3; 28)	23.7 (7.2; 26)	23.9 (7.4; 26.5)	22.0 (8.3; 24)	25.6 (7.4; 29)
Number of wear days per week, mean (SD; median)	5.5 (2.0; 6.5)	6.1 (1.5; 7)	5.8 (1.7; 7)	5.9 (1.5; 7)	5.7 (1.7; 6)	5.7 (1.7; 6)	6.0 (1.6; 7)
Number of wear hours per day, mean (SD; median)	12.4 (3.1; 12.5)	13.8 (4.6; 13.7)	12.6 (5.2; 13.0)	11.7 (4.4; 11.8)	11.0 (4.7; 10.6)	11.0 (4.9; 11.4)	10.0 (5.2; 9.7)

The wear time per day was assessed across all of the months that the socks were worn. The average wear time per day was 11.0 (SD 4.9) hours, and the median was 11.1 hours (Table 2).

In total, 24 people ceased to be active during the studied period; 11 patients temporarily paused their use of the socks, and 13 were lost to follow-up and went off service (Tables 3 and 4).

**Table 3:** Smart sock retention.

Retention characteristics	January 2021	February 2021	March 2021	April 2021	May 2021	June 2021	July 2021
Active patients, n	10	20	23	62	110	133	136
Patients retained (ie, those still active at the end of July), n (%)	9 (90)	17 (85)	20 (87)	52 (84)	95 (86)	122 (92)	N/A^a^

^a^N/A: not applicable.

**Table 4:** Smart sock retention: reasons for inactivity.

Reason	Patients, n
Paused temporarily due to other health conditions (eg, healing from surgery, open wounds, or other health issues)	6
Paused while resolving technical issues	3
Paused while waiting for cooler weather	2
Deceased	1
Changed providers when moving into a permanent nursing home	1
Returns related to comfort	2
Returns due to a lack of education on the use and intent of the device	4
Lost to follow-up and did not respond to repeated calls	5

Patients’ average age at the time of enrollment was 69.9 (SD 10.7; median 71) years. The youngest patient was 37 years old, and the oldest was 94 years old.

## Discussion

### Principal Findings

This study appears to be the first to use data from patients who were being tracked over time during normal pediatric practice to analyze adherence to and compliance with an RPM device for plantar foot temperature. Overall, the temperature monitoring smart socks offered patients with peripheral neuropathy a reliable, easy-to-comply, and real-time device designed to help reduce the risk of foot ulceration. Our findings indicated a high utilization rate of 22 to 25 days per month and a retention rate of 91.9% (147/160; Table 1). The information transmitted from the temperature monitoring smart socks thus allowed providers to closely monitor these high-risk patients.

A number of novel RPM technologies have been developed to provide patients and clinicians with options for monitoring temperature, as temperature is a physiological indicator of inflammation and possibly an early warning sign of foot ulcer formation [4-6]. However, these RPM technologies’ promise for preventing foot ulcers is based on patients’ ability and willingness to use such devices in their activities of daily living. Patients’ compliance with the use of medical devices is known to be poor when it is burdensome to their daily life. A prior study by Armstrong et al [9] found that the utilization rate of using a removable cast walker as an offloading device for DFUs was low [10]. The compliance rate for the offloading device was only as high as 28%. The compliance and utilization rates for the smart socks were considerably higher, despite the average age of the enrollees at the time of enrollment being 69.9 (SD 10.7) years. This suggests that the technology is easy to use, even for older users, who are at increased risk of diabetes and DFUs.

Smart socks, smart pads, and smart insoles are among the RPM devices discussed in the literature [9,11,12]. The reported utilization rate of smart insoles is roughly 6.1 to 6.9 hours per day, and that of the smart pad averages from 1.6 to 4.1 days per week [13,14]. The smart socks in this study reported a high utilization and compliance rate; the socks were used for 22 to 25 days per month and 5.8 days per week on average (Table 2).

The results of this study suggest that the smart sock was used by patients to a high degree, as patients wore the device for an average of 22 to 25 days per month during the period studied. Notably, the percentage of patients that wore the device for at least 15 days in a month ranged from 79.7% (106/133) to 91.9% (125/136) for any particular month (Table 1). The number of patients who wore the RPM device for >5 days per month was high, ranging from 94% (125/133) to 100% (20/20) each month (Table 1). This high number of patients suggests that many patients who fail to achieve >15 days of wear time can be guided to increase their frequency of wear. The wear time per day was, on average, 11.1 hours (Table 2). This suggests that the smart socks were not worn for a brief period but rather were worn extensively throughout the day. Given our findings, the smart socks achieved a high compliance, utilization, and retention rate.

The retention rate was analyzed to be 91.9% (147/160), with only 13 patients dropping out by going off service either through returns or by being lost to follow-up. Further, 11 patients were still on service, but they temporarily paused their use of the socks due to comorbidities or technical issues (Table 4). A total of 160 patients were enrolled, and 149 were still on service by the end of the study period. Patient retention was reviewed by month, and many of the patients in the registry were added in the middle of the study period.

No specific user research was done into the reasons for the high compliance and utilization rates, but a possible reason for these may be that socks are a simple and unintrusive form for an RPM device. Additionally, the lack of charging and regular contact with a nurse for assistance with the RPM services may have also contributed to the high level of utilization. Further analysis, perhaps through patient questionnaires, may provide further insight into the reasons for high utilization. Self-management and the actual utilization of preventative services and devices are important factors for determining health outcomes in chronic conditions. In general, compliance is defined as “the extent to which a person’s behavior coincides with medical advice” [15]. The International Working Group on the Diabetic Foot released guidelines in 2019 that included a recommendation for temperature monitoring and a daily self-inspection of feet for patients at risk of ulceration [16]. The level of compliance to foot care advice has been studied to a limited degree. One study found that only 38.7% of a sample of 331 patients examined their feet 5 to 7 days per week [17]. Adherence to recommendations for foot temperature monitoring has not been extensively studied. One study did demonstrate that a 50% rate of adherence to recording foot temperature resulted in a significantly lower likelihood of developing an ulcer when compared with lower rates of adherence [18]. These findings suggest that adherence may be a challenge with regard to self-management behaviors among patients with diabetes and that adherence is a meaningful factor. In our study, based on the early results of the utilization of the smart socks, patients have a high level of adherence to prescribed advice on wearing smart socks in a real-world setting.

This study also has a few limitations. The period of observation was limited to 7 months, and many patients entered the registry during the middle and later parts of the evaluation period. Further follow-up and a greater number of patients would be necessary to better assess changes in utilization and retention over time.

### Conclusion

The usefulness of temperature monitoring for podiatric patients with limited or no protective sensation has been demonstrated [5,19,20]. The level of adherence to and the utilization of various temperature monitoring devices need further evaluation. This study shows a high level of utilization and compliance for a smart sock remote temperature monitoring device. Further studies with larger patient groups and a longer follow-up period are warranted to better understand the sustained adherence to RPM among patients with diabetes.

## Abbreviations

DFU: diabetic foot ulcer
IRB: institutional review board
RPM: remote patient monitoring

## References

[1] Su D, Michaud TL, Estabrooks P, Schwab RJ, Eiland LA, Hansen G, DeVany M, Zhang D, Li Y, Pagán JA, Siahpush M (2019). Diabetes management through remote patient monitoring: The importance of patient activation and engagement with the technology. Telemed J E Health.

[2] Oikonomidi T, Ravaud P, Cosson E, Montori V, Tran VT (2021). Evaluation of patient willingness to adopt remote digital monitoring for diabetes management. JAMA Netw Open.

[3] Singh N, Armstrong DG, Lipsky BA (2005). Preventing foot ulcers in patients with diabetes. JAMA.

[4] Benbow SJ, Chan AW, Bowsher DR, Williams G, Macfarlane IA (1994). The prediction of diabetic neuropathic plantar foot ulceration by liquid-crystal contact thermography. Diabetes Care.

[5] Armstrong DG, Holtz-Neiderer K, Wendel C, Mohler MJ, Kimbriel HR, Lavery LA (2007). Skin temperature monitoring reduces the risk for diabetic foot ulceration in high-risk patients. Am J Med.

[6] Lavery LA, Higgins KR, Lanctot DR, Constantinides GP, Zamorano RG, Armstrong DG, Athanasiou KA, Agrawal CM (2004). Home monitoring of foot skin temperatures to prevent ulceration. Diabetes Care.

[7] Golledge J, Fernando M, Lazzarini P, Najafi B, Armstrong DG (2020). The potential role of sensors, wearables and telehealth in the remote management of diabetes-related foot disease. Sensors (Basel).

[8] Reyzelman AM, Koelewyn K, Murphy M, Shen X, Yu E, Pillai R, Fu J, Scholten HJ, Ma R (2018). Continuous temperature-monitoring socks for home use in patients with diabetes: Observational study. J Med Internet Res.

[9] Armstrong DG, Lavery LA, Kimbriel HR, Nixon BP, Boulton AJM (2003). Activity patterns of patients with diabetic foot ulceration: patients with active ulceration may not adhere to a standard pressure off-loading regimen. Diabetes Care.

[10] Gordon IL, Rothenberg GM, Lepow BD, Petersen BJ, Linders DR, Bloom JD, Armstrong DG (2020). Accuracy of a foot temperature monitoring mat for predicting diabetic foot ulcers in patients with recent wounds or partial foot amputation. Diabetes Res Clin Pract.

[11] Najafi B, Mohseni H, Grewal GS, Talal TK, Menzies RA, Armstrong DG (2017). An optical-fiber-based smart textile (Smart Socks) to manage biomechanical risk factors associated with diabetic foot amputation. J Diabetes Sci Technol.

[12] Najafi B, Ron E, Enriquez A, Marin I, Razjouyan J, Armstrong DG (2017). Smarter sole survival: Will neuropathic patients at high risk for ulceration use a smart insole-based foot protection system?. J Diabetes Sci Technol.

[13] Abbott CA, Chatwin KE, Foden P, Hasan AN, Sange C, Rajbhandari SM, Reddy PN, Vileikyte L, Bowling FL, Boulton AJM, Reeves ND (2019). Innovative intelligent insole system reduces diabetic foot ulcer recurrence at plantar sites: a prospective, randomised, proof-of-concept study. Lancet Digit Health.

[14] Isaac AL, Swartz TD, Miller ML, Short DJ, Wilson EA, Chaffo JL, Watson ES, Hu H, Petersen BJ, Bloom JD, Neff NJ, Linders DR, Salgado SJ, Locke JL, Horberg MA (2020). Lower resource utilization for patients with healed diabetic foot ulcers during participation in a prevention program with foot temperature monitoring. BMJ Open Diabetes Res Care.

[15] Haynes RB, Sackett D, Taylor DW (1979). Compliance in Health Care.

[16] Bus SA, Lavery LA, Monteiro-Soares M, Rasmussen A, Raspovic A, Sacco ICN, van Netten JJ, International Working Group on the Diabetic Foot (2020). Guidelines on the prevention of foot ulcers in persons with diabetes (IWGDF 2019 update). Diabetes Metab Res Rev.

[17] Neta DSR, da Silva ARV, da Silva GRF (2015). Adherence to foot self-care in diabetes mellitus patients. Rev Bras Enferm.

[18] Lavery LA, Higgins KR, Lanctot DR, Constantinides GP, Zamorano RG, Athanasiou KA, Armstrong DG, Agrawal CM (2007). Preventing diabetic foot ulcer recurrence in high-risk patients: use of temperature monitoring as a self-assessment tool. Diabetes Care.

[19] Yavuz M, Ersen A, Hartos J, Lavery LA, Wukich DK, Hirschman GB, Armstrong DG, Quiben MU, Adams LS (2019). Temperature as a causative factor in diabetic foot ulcers: A call to revisit ulceration pathomechanics. J Am Podiatr Med Assoc.

[20] Frykberg RG, Gordon IL, Reyzelman AM, Cazzell SM, Fitzgerald RH, Rothenberg GM, Bloom JD, Petersen BJ, Linders DR, Nouvong A, Najafi B (2017). Feasibility and efficacy of a smart mat technology to predict development of diabetic plantar ulcers. Diabetes Care.

